# Insensitivity of Paediatric HIV-1 Subtype C Viruses to Broadly Neutralising Monoclonal Antibodies Raised against Subtype B

**DOI:** 10.1371/journal.pmed.0030255

**Published:** 2006-07-18

**Authors:** Elin Solomonovna Gray, Tammy Meyers, Glenda Gray, David Charles Montefiori, Lynn Morris

**Affiliations:** 1AIDS Virus Research Unit, National Institute for Communicable Diseases, Johannesburg, South Africa; **2**Harriet Shezi Clinic, University of the Witwatersrand, Johannesburg, South Africa; **3**Perinatal HIV Research Unit, University of the Witwatersrand, Johannesburg, South Africa; **4**Duke University Medical Centre, Durham, North Carolina, United States of America; National Institute of Child Health and Human DevelopmentUnited States of America

## Abstract

**Background:**

A Phase I clinical trial has been proposed that uses neutralising monoclonal antibodies (MAbs) as passive immunoprophylaxis to prevent mother-to-child transmission of HIV-1 in South Africa. To assess the suitability of such an approach, we determined the sensitivity of paediatric HIV-1 subtype C viruses to the broadly neutralising MAbs IgG1b12, 2G12, 2F5, and 4E10.

**Methods and Findings:**

The gp160 envelope genes from seven children with HIV-1 subtype C infection were cloned and used to construct Env-pseudotyped viruses that were tested in a single-cycle neutralisation assay. The epitopes defining three of these MAbs were determined from sequence analysis of the envelope genes. None of the seven HIV-1 subtype C pseudovirions was sensitive to 2G12 or 2F5, which correlated with the absence of crucial N-linked glycans that define the 2G12 epitope and substitutions of residues integral to the 2F5 epitope. Four viruses were sensitive to IgG1b12, and all seven viruses were sensitive to 4E10.

**Conclusions:**

Only 4E10 showed significant activity against HIV-1 subtype C isolates, while 2G12 and 2F5 MAbs were ineffective and IgG1b12 was partly effective. It is therefore recommended that 2G12 and 2F5 MAbs not be used for passive immunization experiments in southern Africa and other regions where HIV-1 subtype C viruses predominate.

## Editors' Summary

**Background.:**

AIDS is caused by HIV. By killing the cells of the body's immune system, HIV infection makes people vulnerable to many potentially fatal bacterial and viral diseases. HIV is most commonly spread through unprotected sex with an infected partner but it can also pass from mother to child during late pregnancy or birth, or through breast milk. At least one in four infected women will transmit HIV to their babies if left untreated. But if infected women are treated with drugs that fight HIV—so-called antiretrovirals—during late pregnancy and if breastfeeding does not occur, only one to two babies in 100 will become infected with HIV. In addition, elective Caesarian section has been found to be protective against HIV infection. Implementation of this approach has greatly reduced mother-to-child transmission in developed countries, but most HIV-infected women live in developing countries where access to antiretrovirals is limited. In these cases, treatment of pregnant women (during pregnancy and delivery) and their newborn babies with a single dose of one antiretroviral drug, which can halve HIV transmission, is used, even though WHO/UNAIDS recommends simple antenatal, intrapartum, and postnatal antiretroviral regimens to achieve levels of less than 5% transmission in resource poor settings. These strategies will not have an impact on breastmilk transmission, which accounts for half the transmissions in these settings.

**Why Was This Study Done?:**

One way to reduce breastmilk transmission of HIV might be by “passive immunization.” In this, newborn babies would be injected with HIV-specific antibodies—proteins that stick to molecules on the surface of HIV. Because the virus uses these molecules to invade the baby's immune cells, injected antibodies might stop HIV from the mother becoming established in her offspring. Four antibodies have been made in the laboratory—so-called human monoclonal antibodies—that bind to the surface of HIV subtype B, which is found mainly in Europe and North America, and stop HIV from killing human cells. However, most HIV isolated in Africa is subtype C, so in this study researchers have tested whether these antibodies prevent HIV subtype C killing cells grown in the laboratory. It is important, they argue, that antibodies should be shown to work outside the body before testing passive immunization in babies.

**What Did the Researchers Do and Find?:**

The researchers isolated several subtype C viruses from babies born in Johannesburg, South Africa, and made artificial viruses (known as “pseudotyped” viruses) from them. These artificial viruses could then be used in tests to see whether the human monoclonal antibodies could prevent the viruses infecting human cells in a laboratory test, that is, whether the viruses were “sensitive” to the antibodies. All the viruses were insensitive to two of the antibodies (2G12 and 2F5), and the researchers show that this was because the viruses lacked the specific parts of the HIV surface molecules recognized by these antibodies. Four of the viruses were sensitive to an antibody called IgG1b12, and all were sensitive to antibody 4E10, albeit at high concentrations that might be difficult to achieve in people. Finally, the researchers report that the sensitivity of the viruses was not enhanced by using all four antibodies at the same time.

**What Do These Findings Mean?:**

Given these results, the researchers warn against using 2G12 and 2F5 antibodies for passive immunization to prevent mother-to-child transmission, in particular postnatal transmission, in areas where most people are infected with HIV subtype C viruses. Furthermore, because animal studies have indicated that only combinations of at least three monoclonal antibodies with activity against HIV in laboratory tests provide complete protection against HIV infection, the researchers question whether any clinical trials on passive immunization should be started with currently available antibodies. Their doubts about such trials are heightened by observations that 4E10 and 2F5 react against antigens present on human cells, which might make them unsafe for use in people, although so far no adverse effects have been seen in adults treated with these antibodies. However, these experiments used an artificial laboratory-based assay and it's possible that these antibodies might kill HIV subtype C more effectively in people; other components of the immune system might help them deal with the virus. If clinical studies of these antibodies do go ahead, it is essential that the babies in these trials must be carefully monitored to ensure that the antibodies are safe, and they and their mothers should also be given access to optimal antiretroviral prophylaxis according to WHO/UNAIDS guidelines. In a related *PLoS Medicine* Perspective paper (http://dx.doi.org/10.1371/journal.pmed.0030259), Miroslaw Gorny1 and Susan Zolla-Pazner discuss the study further and stress the critical need to determine if passive immunization with such antibodies could decrease mother-to-child transmission of HIV, and if so what the best antibodies would be.

**Additional Information.:**

Please access these Web sites via the online version of this summary at http://dx.doi.org/10.1371/journal.pmed.0030255.

• National Institute of Allergy and Infectious Diseases fact sheets on HIV infection and AIDS

• US Department of Health and Human Services information on HIV/AIDS, including clinical guidelines and fact sheets on preventing transmission from mother to child

• US Centers for Disease Control and Prevention information on HIV/AIDS, including pages on the prevention of mother-to-child transmission

• MedlinePlus encyclopedia entry on HIV/AIDS

• Preventing mother-to-child transmission of HIV Web page

## Abstract 3

Assessment of viruses from seven children with HIV-1 subtype C infection showed generally poor sensitivity to four monoclonal antibodies proposed for a trial of passive immunoprophylaxis to prevent mother-to-child transmission.

## Introduction

Only four broadly neutralising monoclonal antibodies (MAbs) against HIV-1 have been generated to date, all of which were derived from patients with HIV-1 subtype B infection. IgG1b12 recognizes an epitope overlapping the CD4 binding site in the envelope glycoprotein complex [ 1– 5], and 2G12 recognizes a mannose-rich epitope on the silent face of gp120 [ 6– 10]. The 2F5 and 4E10 linear MAbs are located in the membrane-proximal external region of gp41 [ 11– 13]. Passive transfer studies in primates using combinations of these MAbs have provided strong evidence that MAbs are able to control viral replication [ 14– 17] and prevent HIV-1 infection parenterally and through mucosal tissues [ 18, 19]. More recent data have shown that in some individuals with HIV infection, these MAbs can reduce the rate of viral rebound following a structured treatment interruption [ 20]. Furthermore, oral challenge studies in neonatal macaque monkeys support the use of neutralising MAbs for prevention of virus transmission to human infants [ 21, 22].

Mother-to-child transmission (MTCT) of HIV-1 infection remains a significant problem in developing countries. While the use of single-dose nevirapine, acting to prevent intrapartum transmission, has reduced the number of infections, more potent interventions are needed, particularly to prevent postpartum transmissions. It is estimated that in South Africa alone, approximately 96,000 children with HIV-1 infection were born in 2003 [ 23]. Passive immunization using neutralising MAbs has been suggested as a strategy to prevent breast milk–borne infections [ 24, 25]. Whether this approach is valid is likely to depend on the efficacy of these MAbs against the targeted viruses.

The most common subtype of HIV-1 infection in southern Africa as well as globally is subtype C ( http://www.unaids.org). Results from a previous study indicated that a combination of the MAbs 2F5, 2G12, IgG1b12, and 4E10 successfully neutralised 100% of HIV-1 subtype C isolates tested [ 26]. However, other studies have shown that 2F5 and 2G12 MAbs are usually ineffective against HIV-1 subtype C viruses, while 4E10 is able to neutralise isolates from all subtypes [ 27, 28]. To further address whether 2G12, 2F5, IgG1b12, and 4E10 are active against HIV-1 subtype C viruses, we tested them in an Env-pseudotyped virus infectivity assay. We chose to use specifically those viruses derived from infants and children who had perinatally acquired HIV-1 infection to determine whether or not these MAbs are effective in vitro as an indication of their potential use for prevention of MTCT.

## Methods

### HIV-1 Subtype C Viral Isolates

Viruses were isolated from the blood of children with HIV-1 infection by standard co-culture techniques using peripheral blood mononuclear cells (PBMCs) [ 29, 30]. Blood samples were collected from children residing in an orphanage or receiving medical care at the Chris Hani Baragwanath Hospital in Johannesburg between 1999 and 2002 ( Table 1) [ 29]. Informed consent was obtained from either a parent or a guardian of each child at the time of blood collection. This study received ethical approval from the University of the Witwatersrand Committee for Research on Human Subjects (Medical) (Johannesburg, South Africa).

**Table 1:**
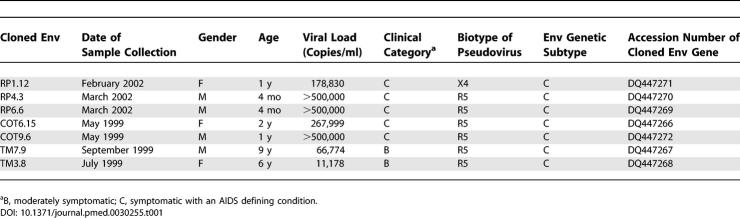
Patient Information and Viral Isolate Characteristics for HIV-1 Subtype C Cloned Envelope Genes

### MAbs, sCD4, and Plasma Samples

MAbs were obtained from the National Institutes of Health Reference and Reagent Program (Germantown, Maryland, United States) and the International AIDS Vaccine Initiative Neutralizing Antibody Consortium (New York, New York, United States), and used at a starting concentration of 50 μg/ml. Recombinant soluble CD4 (sCD4) comprising the extracellular domain of human CD4 produced in Chinese hamster ovary cells was obtained from Progenics Pharmaceuticals (Tarrytown, New York, United States), and tested at 50 μg/ml. Two plasma samples (BB12 and IBU21) from blood donors with HIV-1 subtype C infection were tested at a starting dilution of 1:50.

### Cell Lines

JC53-bl cells were obtained from the National Institutes of Health Reference and Reagent Program (catalog number 8129). These cells were derived from a HeLa cell clone that expresses CD4, CCR5, and CXCR4 constitutively [ 31] and contains two reporter genes: firefly luciferase and Escherichia coli β-galactosidase under the control of the HIV-1 LTR promoter [ 32]. The 293T cells used for transfection were obtained from the American Type Culture Collection (Manassas, Virginia, United States). Both cell lines were cultured in D-MEM containing 10% heat-inactivated fetal bovine serum. Cell monolayers were disrupted at confluency by treatment with 0.25% trypsin in 1mM EDTA.

### Cloning of Envelope Genes and Production of Pseudovirions

Proviral DNA extracted from in vitro infected PBMCs was used to amplify full-length envelope genes. The 3-kilobase PCR fragments, generated using envA and envM primers [ 33], were cloned into the pCDNA 3.1-TOPO vector (Invitrogen, Carlsbad, California, United States) and bacterial colonies screened by PCR for insertion and correct orientation using T7 and envM primers. The Env-pseudotyped virus stocks were generated by co-transfecting 2 μg of the *env* encoding plasmid DNA with 3.3 μg of the HIV genomic vector SG3delta *env* (a gift from Beatrice Hahn) into an 80% confluent monolayer of 293T cells in a T-25 culture flask in the presence of 40 μl of PolyFect Transfection Reagent (Qiagen, Heidelberg, Germany). The media was replaced 6–8 h after transfection; 48 h later, culture supernatant containing the pseudoviruses was harvested, filtered (0.45 μm), and stored at −70 °C. The tissue culture infectious doses (TCID _50_) were quantified by infecting JC53-bl cells with serial 5-fold dilutions of the supernatant in quadruplicate in the presence of DEAE dextran (30 μg/ml) (Sigma, St. Louis, Missouri, United States). The infection was monitored 48 h later by evaluating the luciferase activity using the Bright Glo Reagent (Promega) following manufacturer instructions. Luminescence was measured in a Wallac 1420 Victor Multilabel Counter (Perkin Elmer, Wellesley, California, United States). TCID _50_ was calculated as described [ 34]. Wells with relative light units greater than 2.5 times the negative control (mock infection) were considered positive for infection.

### Single-Cycle Neutralisation Assay

Neutralisation was measured as a reduction in luciferase gene expression after a single-round infection of JC53-bl cells with Env-pseudotyped viruses [ 35]. Briefly, 200 TCID _50_ of pseudoviruses in 50 μl culture media was incubated with 100 μl of serially diluted MAbs, plasma, or sCD4 using D-MEM with 10% fetal bovine serum in a 96-well plate in triplicate for 1 h at 37 °C. MAbs were either tested singly starting at 50 μg/ml (before addition of cells) or in combination also at 50 μg/ml for each MAb. Thus, TriMab contained 2G12, IgG1b12, and 2F5 (50:50:50 μg/ml) and TriMab plus 4E10 contained 2G12, IgG1b12, 2F5, and 4E10 (50:50:50:50 μg/ml). A 100-μl solution of JC53-bl cells (1 × 10 ^4^ cells/well) containing 75 μg/ml DEAE dextran was added; the cultures were then incubated at 37 °C in 5% CO _2_/95% air for 48 h. Infection was monitored by evaluating the luciferase activity. Titres were calculated as inhibitor concentration (IC _50_) or reciprocal plasma dilution (ID _50_) values causing 50% reduction of relative light units compared to the virus control (wells with no inhibitor) after subtracting the background (wells without virus infection). IC _50_ values obtained for MAb combinations were compared to MAbs tested singly. The HIV-1 subtype B pseudovirus QH692.42 was included as a positive control, because this virus has been known to be sensitive to all four of the test MAbs [ 36, 37].

### gp160 Sequencing

Cloned *env* genes were sequenced using the ABI PRISM Big Dye Terminator Cycle Sequencing Ready Reaction kit (Perkin-Elmer Applied Biosystems, Foster City, California, United States) and resolved on an ABI 3100 automated genetic analyzer. The full-length gp160 sequences were assembled and edited using Sequencher (version 4.0) software (Gene Codes, Ann Arbor, Michigan, United States).

## Results

### HIV-1 Subtype C Cloned Envelopes from Paediatric Patients

We cloned complete (gp160) envelope genes from seven HIV-1 subtype C isolates cultured from the blood of children with perinatally acquired HIV-1 infection. Five of these isolates were from rapidly progressing infants (RP and COT) who developed severe clinical symptoms within the first year of life, most of whom died shortly after blood collection ( Table 1). Two isolates were from children who had survived for between 6 and 9 y and were moderately symptomatic with illnesses, such as lymphocytic interstitial pneumonitis. All isolates used the CCR5 co-receptor, while viruses from one rapidly progressing infant (RP1) also used the CXCR4 co-receptor and was therefore dualtropic [ 29]. The Env-pseudotyped virus derived from the latter isolate was able to use only CXCR4 as co-receptor, while the other six pseudoviruses used CCR5 ( Table 1). All cloned envelopes were sequenced and compared to the original viral isolate. Phylogenetic analysis indicated that all samples were HIV-1 subtype C isolates; sequences from the same individual clustered together with high bootstrap values (unpublished data).

### Neutralisation Sensitivity of HIV-1 Subtype C Env-Pseudotyped Viruses to MAbs

The HIV-1 subtype C envelope clones were used to generate Env-pseudotyped viruses by co-transfection with a subgenomic plasmid. These pseudoviruses were tested for their sensitivity to neutralisation by the MAbs IgG1b12, 2G12, 2F5, and 4E10. The MAbs 2G12 and 2F5 failed to neutralise any of the seven HIV-1 subtype C pseudoviruses at 50 μg/ml, whereas the HIV-1 subtype B virus QH692.42 had IC _50_ values of 0.8 and 7.1, respectively ( Table 2). The IgG1b12 neutralised four of the seven HIV-1 subtype C viruses as well as the HIV-1 subtype B control. The IC _50_ values of the sensitive pseudoviruses ranged from 0.2 μg/ml to 12 μg/ml, indicating high potency of this MAb. The MAb 4E10 neutralised all the viruses. The IC _50_ values were generally high, supporting the notion that this MAb has broad specificity but lower potency than other MAbs [ 27].

**Table 2:**
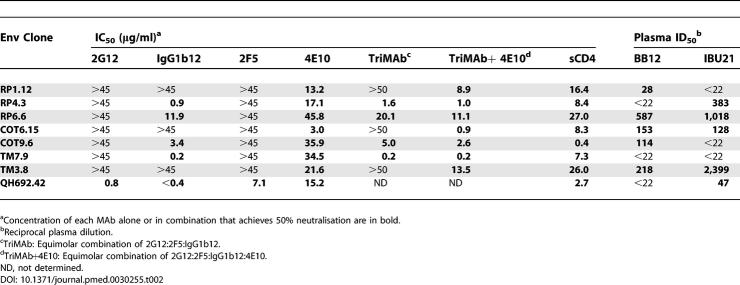
Sensitivity of HIV-1 Subtype C Pseudovirions to Anti-HIV MAbs, sCD4, and Plasma

### Neutralisation Using Combinations of MAbs

Synergistic neutralisation among MAbs that recognize different specificities in the envelope glycoprotein has been suggested [ 38, 39], although it has been a controversial topic. We decided, therefore, to test combinations of these MAbs using equimolar concentrations of 2G12, IgG1b12, and 2F5 (TriMAb), and TriMAb plus 4E10. The IC _50_ values in the presence of TriMAb were similar to those for IgG1b12 alone ( Table 2), indicating that the activity in TriMAb was probably due to the activity of IgG1b12. When 4E10 was added to TriMAb, it was not surprising that neutralisation of all isolates was achieved, because 4E10 was active against all isolates when used alone at this concentration range.

Analysis of the dose-response curves confirmed the lack of significant synergy among MAbs. Those viruses sensitive to IgG1b12 (RP4.3, RP6.6, TM7.9, and COT9.6) had similar neutralisation curves in the presence of IgG1b12 alone or when tested as part of TriMAb with or without 4E10 ( Figure 1A). However, among isolates insensitive to IgG1b12 (COT6.15, TM3.8, and RP1.12), slightly greater potency was observed with TriMAb plus 4E10, compared to 4E10 alone ( Figure 1B).

**Figure 1:**
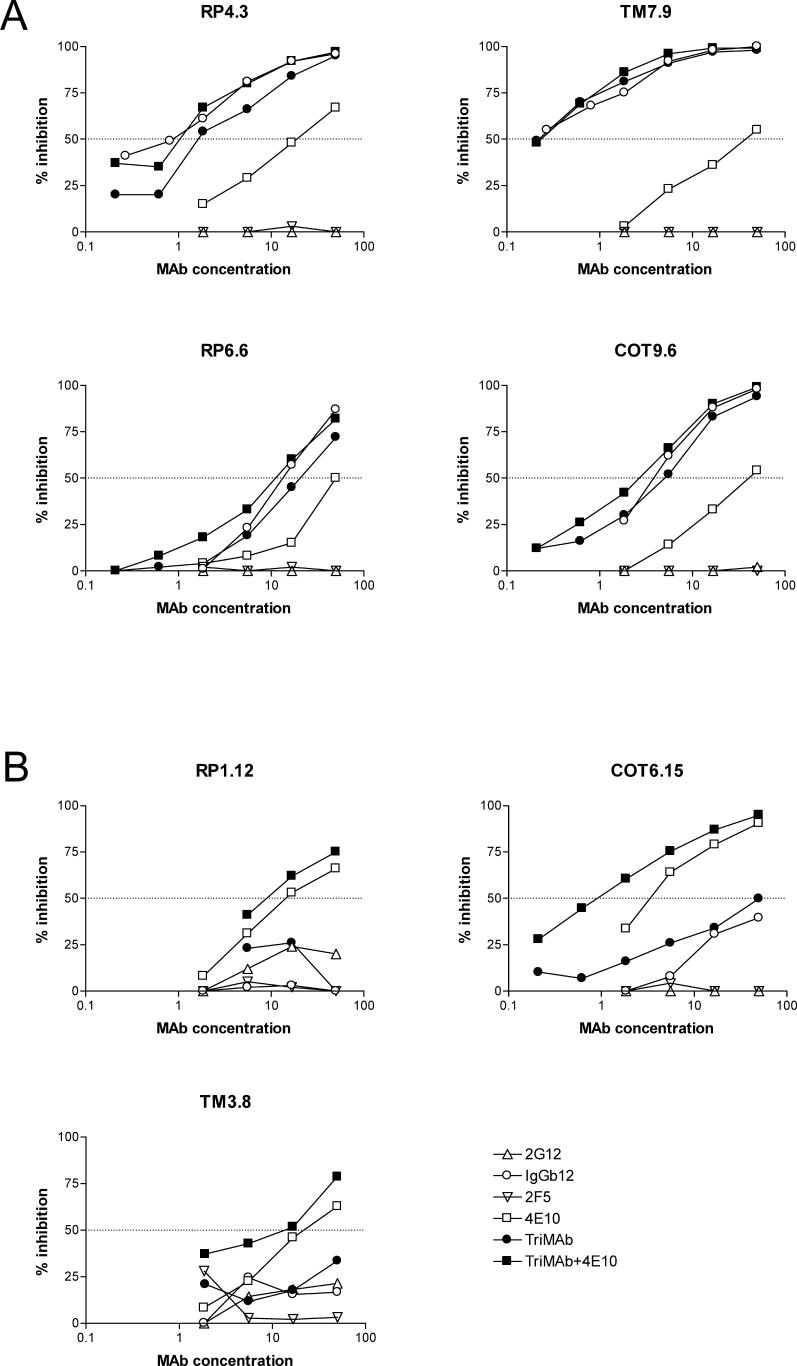
Neutralisation Dose-Response Curves of the MAbs 2G12, 2F5, IgG1b12, and 4E10, Alone and in Combination The MAb concentrations in the triple and quadruple combination are represented as the concentration of each MAb in the equimolar mix starting at 50 μg/ml. Results are shown as the reduction of virus infectivity relative to the virus control (without MAbs) with 50% inhibition indicated by a dotted line. Note those viruses sensitive to IgG1b12 and 4E10 (A) and those viruses sensitive to 4E10 alone (B).

### Sensitivity to sCD4 and Polyclonal Anti-HIV Antibodies

Given the relative resistance of the HIV-1 subtype C pseudovirions to neutralisation by MAbs, we chose to test their responses to sCD4 and polyclonal antibodies from individuals with HIV-1 infection. sCD4, which blocks gp120 binding to the CD4 receptor, neutralised all of the pseudovirions ( Table 2), indicating that the CD4 binding site is accessible on the pseudotyped envelope glycoproteins. The IgG1b12 binding site overlaps with the CD4 binding site; however, there was no correlation between the ID _50_ values for sCD4 and IgG1b12 in this assay, similar to what others have reported [ 28, 40].

All pseudovirions except TM7.9 were neutralised by one or both of the plasma samples with a wide variation in IC _50_ titres, as is often seen when using polyclonal antibodies, suggesting that these envelopes were not atypical in their ability to be neutralised ( Table 2).

### Analysis of Amino Acid Sequences Comprising the Neutralisation Epitopes

Sequence analysis of the predicted N-linked glycosylation (PNG) sites at positions 295, 332, and 392, which are critical for the 2G12 epitope, indicated that all HIV-1 subtype C isolates lacked the glycan 295. TM7.9 also lacked the glycan 392 ( Table 3). Another site (position 386), reported to play an indirect role in the formation of the 2G12 epitope, was also absent from one of the HIV-1 subtype C envelopes. The HIV-1 subtype B pseudovirus QH692.42 was the only virus possessing all five PNG sites and was the only virus sensitive to 2G12. These data suggest that the lack of the glycan 295 renders isolates resistant to 2G12, as previously suggested [ 9, 10].

**Table 3:**
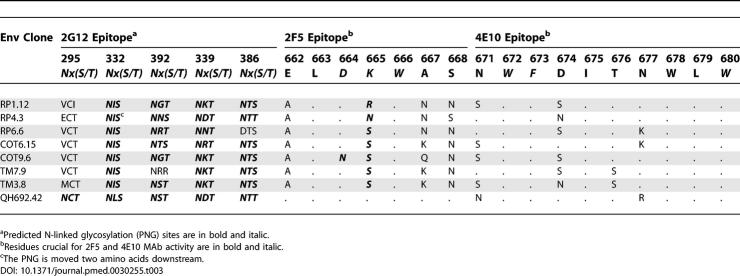
Amino Acid Sequences of MAb Epitopes in Cloned Subtype C Envelope Genes

The 2F5 epitope is centred on the sequence ELDKWA [ 11]. Mutagenesis studies have revealed that the amino acid residues DKW are indispensable for the recognition by this MAb [ 13, 41]. In particular, substitutions at residue K665 appear to be the major determinant of resistance [ 27]. In this study, all HIV-1 subtype C isolates had substitutions at position 665 with the lysine (K) residue replaced by serine (S) or other amino acids (R or N), while the HIV-1 subtype B pseudovirus QH692.42 had no such substitution. These data support the finding that the residue K665 is crucial for neutralisation by 2F5.

4E10 recognizes an epitope containing the sequence NWF(D/N)IT [ 12, 42] at the C-terminal of the 2F5 epitope. Mutagenesis experiments have shown that the residues W672, F673, and W680 are indispensable for recognition by 4E10 [ 13], while the crystal structure of the Fab 4E10-epitope complex indicates that W672, F673, I675, and T676 are the key residues in this interaction [ 43]. All the viruses analyzed in this study had a conserved 4E10 epitope (W672, F673, W680), consistent with their phenotypic sensitivity to this MAb.

## Discussion

The neutralisation sensitivity of HIV-1 subtype C isolates derived from children appears similar to previously reported sensitivity of isolates from adults with HIV-1 subtype C infection [ 27, 28]. Thus the broadly cross-reactive neutralising MAbs 2G12 and 2F5 are ineffective against both paediatric and adult HIV-1 subtype C viruses, while IgG1b12 potently neutralised approximately 50% of the tested viruses. Only 4E10 showed broad activity against HIV-1 subtype C viruses, although its potency was low. Collectively, these data caution against the use of 2G12 and 2F5 MAbs for passive immunization in areas where HIV-1 subtype C viruses are highly prevalent.

In this study, we have used cloned envelope genes in a single-cycle neutralisation assay, which is a high-throughput assay that, to our knowledge, is rapidly becoming the method of choice for measuring antibody neutralisation [ 37, 44]. Comparative studies have shown a positive correlation between results derived from this assay and the more traditional PBMC-based neutralisation assay (Taylor et al., unpublished data) [ 37]. However, the 293T-derived pseudovirions were found to be more sensitive to neutralisation by MAbs and serum samples when compared to the uncloned PBMC-derived viruses [ 27, 37]. It has been suggested that this effect is due to the cells used to generate the pseudoviruses [ 44] and not the nature of the target cells or the clonal nature of the envelope [ 27, 45]. Overall, we can be confident that the observed resistance of HIV-1 subtype C isolates to 2G12 and 2F5 is not due to the use of an Env-pseudotyped virus-based neutralisation assay. Instead, the extra sensitivity of the latter assay might be expected to generate false-positive and not false-negative outcomes.

It has been shown in multiple studies that 2G12 is generally ineffective against HIV-1 subtype C isolates [ 27, 28]. The 2G12 epitope binds a cluster of mannose residues; the absence of an N-linked glycan at position 295 appears to correlate with resistance to this MAb [ 9, 10]. The absence of N295 may prevent the correct processing and presentation of glycans at position 332, affecting antibody binding and therefore neutralisation [ 7]. A recent study has shown that reintroduction of this PNG site into a subtype C isolate restored binding of 2G12, although sensitivity to neutralisation was not tested [ 46]. An analysis of 339 HIV-1 subtype C envelope sequences obtained from Los Alamos Database showed that 83% of sequences lacked a glycosylation site at position 295. If the lack of the PNG at position 295 is indeed a cause of resistance to 2G12, then a majority of HIV-1 subtype C viruses would be insensitive to this MAb.

The 2F5 MAb has been shown to have broadly neutralising activity but has minimal efficacy against HIV-1 subtype C viruses [ 27, 28]. An alanine scan over the ELDKWAS epitope defined the motif DKW in positions 664–666 as a determinant for 2F5 recognition [ 13], although some viruses with this epitope are insensitive to this MAb [ 27]. However, all viruses with a substitution at residue K665 are resistant to 2F5 [ 27]. Similarly, in this study, we found that all resistant viruses had a substitution at K665 while the subtype B virus did not. Analysis of 324 sequences in Los Alamos Database showed that the subtype C consensus for the 2F5 epitope is ALDSWA, with only approximately 12% bearing a K at position 665. This suggests that the majority of HIV-1 subtype C viruses will also be resistant to 2F5. However, a geographical clustering of some HIV-1 subtype C variants that may be sensitive to 2F5 due to the presence of the DKW epitope has been suggested [ 27].

Our data with IgG1b12 agree with other studies in that this MAb is more effective than 2F5 or 2G12 at neutralising HIV-1 subtype C viruses, although IgG1b12 inhibited only approximately 50% of the isolates tested [ 27, 28]. Among sensitive isolates, this MAb is particularly potent and requires very low antibody concentrations for 50% inhibition. Due to the conformational nature of the IgG1b12 epitope, it is difficult to predict resistance to this MAb by simple sequence analysis. Some studies have described neutralisation escape mutations for this MAb, such as a proline-to-alanine substitution in position 369 in the C3 region of gp120 [ 47, 48]. In this study, we found no correlation between the presence of a proline at this position and sensitivity to IgG1b12, which suggests that this escape mutation was specific to the isolate used in the referred study.

The 4E10 epitope appears to be the most broadly cross-reactive MAb described to date, neutralising all viruses so far tested. In previous studies, 4E10 has been shown to neutralise 100% of viruses in a comprehensive panel that included all genetic subtypes of HIV-1 group M and some recombinant forms [ 27, 49]. However, 4E10 is a low-potency antibody generally requiring high concentrations to reduce infectivity by 50%, as seen in this and other studies [ 20, 27]. Whether this is a property of the antibody or inaccessibility of the epitope remains to be determined. The motif WF on the 4E10 epitope was 100% conserved in 324 sequences of this portion of gp41 from HIV-1 subtype C viruses in Los Alamos Database. This suggests that HIV-1 subtype C viruses will be universally sensitive to 4E10.

Some studies have suggested that MAbs can act synergistically to increase neutralisation potency against HIV-1 [ 26, 38, 39, 50, 51]. However, this has been a controversial topic, and isolate dependency has been observed [ 51, 52] with different results obtained with T-cell line-adapted virus and primary isolates [ 39, 50, 52]. In this study, we did not observe strong synergy among these MAbs. The combination of the four MAbs neutralised all the tested viruses in agreement with other study results for HIV-1 subtype C isolates [ 26]. This is likely due to the neutralisation activity of individual MAbs rather than the combined effect of them, because a significant increase in potency was not observed with the mixtures. There may have been a slight synergistic effect for RP1.12, TM3.8, and COT6.15 as demonstrated by increased neutralisation when 4E10 was combined with IgG1b12, 2F5, and 2G12. Such an effect is probably due to IgG1b12, given the absence of the 2G12 and 2F5 epitopes in these viruses. A more thorough analysis of synergism would require titrating 4E10 against IgG1b12 and evaluating the data based on the Chou-Talalay method [ 39, 53]. It is also possible that the clonal nature of the envelope glycoproteins used in this study precluded the detection of synergism. Some researchers have suggested that the heterogeneity of the virus is the cause of the synergistic effects of some neutralising antibody combinations [ 54]. However, others have observed no differences between virus isolates passaged in PBMCs and cloned envelope pseudotype viruses [ 39].

The MTCT of HIV-1 infection is usually associated with transmission of single variants [ 26]. In this study, four of the cloned envelopes were from children infected for fewer than 12 mo, two of which were infected for 4 mo and therefore represent relatively early variants. Although these clones may not have been the earliest transmitted variants, it is unlikely that earlier variants would differ in their neutralisation sensitivity to these MAbs. We base this assumption on the fact that the MAb sensitivities of viruses from adults with HIV-1 subtype C infection, who would be the source of infection in perinatally infected children, are similar. In addition, we did not observe variation in the susceptibility to neutralisation or in the epitope sequences that can be related to the age of the child: infants and children in this study had identical phenotypic and genotypic profiles. Overall, we feel confident that the MAb neutralisation profiles of the viruses analyzed in this study would be representative of the earliest transmitted variants.

Based on our results, we question the use of MAb combinations that include 2F5 and 2G12 as a prophylactic treatment in regions where HIV-1 subtype C viruses predominate, even if such combinations were to include 4E10 and IgG1b12. In passive immunoprophylaxis studies using a single MAb, protection was not observed even when the challenge strain was successfully neutralised in vitro. Only a combination of at least three MAbs with bona fide neutralisation activity against the challenge strain offered complete protection [ 17]. Such a combination is not likely to be achievable against HIV-1 subtype C isolates. Furthermore, a recent study using a combination of 2G12, 2F5, and 4E10 for the treatment of individuals with HIV-1 infection has denoted that the ability of 2F5 and 4E10 to affect the virus in vivo is unclear and may require very high serum concentrations of these MAbs [ 20]. This further questions the use of MAb combinations in which only 4E10 has the potential to be 100% effective.

Overall, we believe that the use of these MAbs to prevent MTCT of HIV-1 subtype C infection is unlikely to be efficacious; therefore, a clinical trial should not be conducted. A recent study has confirmed our viewpoint that these MAbs would have limited benefit when used to prevent MTCT in populations with HIV-1 non-B subtype infection [ 55]. In addition, recent data have suggested that the MAbs 2F5 and 4E10 react against self-antigens, such as cardiolipin, and the MAb IgG1b12 reacts with double-stranded DNA [ 56]. Although safety concerns exist surrounding the use of these MAbs for treatment [ 57], no adverse effects have yet been reported in treated adults [ 20]. It should be noted that this work remains to be corroborated by others. Nevertheless, if these findings on autoreactivity prove to be true, then the utility of these MAbs for in vivo use is in further doubt, particularly if they are to be used in infants.

The study of the epitopes recognized by these broadly neutralising MAbs contributes to the knowledge necessary for the rational design of an immunogen capable of inducing a broad and potent neutralisation response against HIV-1 infection. Considerable efforts have been invested in designing immunogens based on these epitopes [ 41]. However, given the subtype constraints of some of these epitopes, new, more broadly occurring epitopes need to be found for the design of vaccines that will be able to elicit an efficient neutralising response against a broad spectrum of HIV subtypes.

## Supporting Information

### Accession Numbers

The GenBank ( http://www.ncbi.nlm.nih.gov/Genbank) accession numbers for nucleotide sequences of the cloned envelope genes discussed in this paper are DQ447266–DQ447272 ( Table 1).

## Abbreviations

IC _50_: inhibitor concentration
MAb: monoclonal antibody
MTCT: mother-to-child transmission
PBMC: peripheral blood mononuclear cell
PNG: predicted N-linked glycosylation
sCD4: soluble CD4
TCID _50_: tissue culture infectious dose
